# A Process-Oriented View of Procedural Memory Can Help Better Understand Tourette’s Syndrome

**DOI:** 10.3389/fnhum.2021.683885

**Published:** 2021-12-10

**Authors:** Bence Cs. Farkas, Eszter Tóth-Fáber, Karolina Janacsek, Dezso Nemeth

**Affiliations:** ^1^LNC^2^, Département d’Études Cognitives, École Normale Supérieure, INSERM, PSL Research University, Paris, France; ^2^Doctoral School of Psychology, ELTE Eötvös Loránd University, Budapest, Hungary; ^3^Institute of Psychology, ELTE Eötvös Loránd University, Budapest, Hungary; ^4^Brain, Memory and Language Research Group, Institute of Cognitive Neuroscience and Psychology, Research Centre for Natural Sciences, Budapest, Hungary; ^5^Centre for Thinking and Learning, Institute for Lifecourse Development, School of Human Sciences, Faculty of Education, Health and Human Sciences, University of Greenwich, London, United Kingdom; ^6^Lyon Neuroscience Research Center (CRNL), INSERM U1028, CNRS UMR 5292, Université Claude Bernard Lyon 1, Lyon, France

**Keywords:** Tourette’s syndrome, procedural memory, basal ganglia, sequence learning, statistical learning, atypical development, neuropsychology, serial reaction time task

## Abstract

Tourette’s syndrome (TS) is a neurodevelopmental disorder characterized by repetitive movements and vocalizations, also known as tics. The phenomenology of tics and the underlying neurobiology of the disorder have suggested that the altered functioning of the procedural memory system might contribute to its etiology. However, contrary to the robust findings of impaired procedural memory in neurodevelopmental disorders of language, results from TS have been somewhat mixed. We review the previous studies in the field and note that they have reported normal, impaired, and even enhanced procedural performance. These mixed findings may be at least partially be explained by the diversity of the samples in both age and tic severity, the vast array of tasks used, the low sample sizes, and the possible confounding effects of other cognitive functions, such as executive functions, working memory or attention. However, we propose that another often overlooked factor could also contribute to the mixed findings, namely the multiprocess nature of the procedural system itself. We propose that a process-oriented view of procedural memory functions could serve as a theoretical framework to help integrate these varied findings. We discuss evidence suggesting heterogeneity in the neural regions and their functional contributions to procedural memory. Our process-oriented framework can help to deepen our understanding of the complex profile of procedural functioning in TS and atypical development in general.

## Habits, Procedures, and Symptoms

Even though humans possess a unique capacity for complex reasoning, a large portion of our day to day behavior is not governed by such higher order, deliberative control. Instead, it is automatic, stimulus driven and less demanding of our cognitive resources; in other words, habitual (Wood and Rünger, 2016). The distinction between goal-directed and habitual control and their respective computational, neurobiological, psychological and behavioral correlates is a topic of intense current research in the emerging fields of neuroeconomics and computational psychiatry (Dolan and Dayan, 2013; O’Doherty et al., 2017). It is now clear that while habits can make us act quickly and efficiently, they can also be harmful. Once they are established, it is hard to change them, even in cases when we recognize them as being maladaptive. Even more importantly, some psychiatric symptoms, like the obsessions and compulsions of Obsessive-Compulsive Disorder (OCD) and addiction can be viewed as extreme manifestations of habitual control over behavior. Certain psychiatric and neurodevelopmental disorders could thus potentially be characterized by a shift in the balance between habitual and goal-directed control (Maia and Frank, 2011). In this short review, we take a closer look at habit learning in one of these disorders, Tourette’s syndrome. For most of this article, we approach habits not from a reinforcement learning but from a complementary, memory systems perspective (Doll et al., 2015). Our reasoning for limiting this review to procedural memory and not discussing other, related perspectives in detail, such as reinforcement learning or declarative memory is twofold. On the one hand, the nature of the relationship between procedural memory and these other possible perspectives is an important and undetermined question in itself (Henke, 2010; Graybiel and Grafton, 2015) and beyond the scope of our review. On the other hand, procedural memory is also the construct that has received the most attention, and has the most published studies to summarize.

We will argue that the basal ganglia-based procedural learning and memory system underlying habits and skills is better understood as a bundle of interacting learning processes, rather than a homogenous system. We discuss and offer a selective review of the complex pattern of findings in Tourette’s syndrome, with studies indicating both normal, impaired and enhanced procedural learning/memory performance. We propose that a process-oriented view of procedural memory functions could serve as a theoretical framework and new perspective to help integrate these varied findings. There is ample evidence from statistical and procedural learning studies for the heterogeneity of computations involved in the learning of perceptual, motor and cognitive skills (Thiessen et al., 2013; Beukema and Verstynen, 2018; Kóbor et al., 2018; Conway, 2020). Additionally, neural evidence indicates both within-task and between-task variability in the basal ganglia and cortical regions underlying procedural learning performance (Doyon et al., 2009; Reber, 2013; Frost et al., 2015). Furthermore, previous studies yielded rather low correlations between procedural learning measures (Marsh et al., 2005; West et al., 2018), suggestive of different sets of mechanisms affecting performance in different tasks. With all of this in mind, we begin our review by briefly introducing the constructs of procedural memory and Tourette’s syndrome and their connection. After this, we discuss our proposition for a process-based approach and the evidence in support of it, in more detail.

The idea that memory is not a unitary construct and a number of different memory systems exist in the mammalian brain is now well accepted in cognitive neuroscience (Squire and Zola, 1996; Gabrieli, 1998). One key distinction in this framework is between declarative and non-declarative memory, the former associated with medial prefrontal and medial temporal lobe structures and conscious awareness (Eichenbaum, 2006), the latter with various cortico-basal ganglia and cortico-cerebellar circuits and the absence of conscious awareness (Reber, 2013). Within non-declarative memory, the basal ganglia based system is responsible for the learning and storage of cognitive, motor and perceptual habits and skills is known as procedural memory (Knowlton et al., 1996; Ullman, 2015; Ullman et al., 2020). There has been an enormous interest in characterizing the functioning of this system in both healthy and clinical populations, including in Tourette’s syndrome (Goodman et al., 2014). We are concerned primarily with this memory system and the possibility of decomposing it into subprocesses for more precise clinical research.

The investigation of procedural memory in order to understand and explain neurodevelopmental disorders has been fruitful (reviewed in Ullman et al., 2020). Robust procedural learning deficits have been found in various disorders of language, including Developmental Dyslexia (Lum et al., 2013; Clark and Lum, 2017) and Developmental Language Disorder (Lum et al., 2014; Clark and Lum, 2017). However, results from neurodevelopmental disorders not primarily affecting language have not universally indicated impairments. For example, procedural learning performance has been found to be intact both in Autism Spectrum Disorder (ASD) (Barnes et al., 2008; Brown et al., 2010; Nemeth et al., 2010; Travers et al., 2010) and Attention Deficit Hyperactivity Disorder (ADHD) (Karatekin et al., 2009; Vloet et al., 2010; Takács et al., 2017; Pedersen and Ohrmann, 2018). Additionally, as we discuss below, the picture is unclear in Tourette’s syndrome as well.

## Tourette’s Syndrome

Tourette’s syndrome (TS) is a neurodevelopmental disorder, characterized by repetitive movements and vocalizations, also known as tics (Robertson, 2000). The prevalence of the condition has been estimated to be around 0.85%, with lower rates in adulthood and higher rates in males (Robertson, 2015). No specific genes have been reliably linked to the disorder but studies indicate a significant genetic contribution to its etiology (Scharf et al., 2013). The most frequent comorbid disorders include ADHD, OCD, and ASD (Robertson, 2015). Symptoms usually start well before puberty and reach their peak around the age of 12, with substantial decline during adolescence. About one third of patients stop ticcing almost entirely by adulthood (Bloch and Leckman, 2009). The tics are commonly preceded by premonitory urges and can be effortfully suppressed, although such suppression is mostly only temporary (Cohen and Leckman, 1992).

Both the neurobiological underpinnings and behavioral symptoms of TS point to a role of procedural memory. On the behavioral level, tics share many commonalities with habits, as both are automatically executed, stereotyped actions that are hard to inhibit, even if they are clearly perceived as maladaptive. These types of behaviors are thought be acquired through procedural learning (Yin and Knowlton, 2006). Furthermore, one of the most effective behavioral interventions in the disorder is habit reversal therapy, which heavily relies on establishing cognitive, goal-directed control over habitual behaviors (Wilhelm et al., 2003). On the neurobiological level, the cortico-basal ganglia-thalamo-cortical (CBGTC) loops seem to have both functional and structural abnormalities in the disorder (Felling and Singer, 2011; Ganos et al., 2013). Striatal volumes have been shown to be altered in TS patients, with studies demonstrating decreased caudate (Peterson et al., 2003; Bloch et al., 2005) and increased putamen volumes (Roessner et al., 2011). Microstructural changes in the putamen, along with the thalamus, have also been observed with Diffusion Tensor Imaging, with increased diffusivity in both structures (Makki et al., 2008). Multiple studies also reported reductions in the number of and a change in the distribution of striatal interneurons (Kalanithi et al., 2005; Kataoka et al., 2010; Rapanelli et al., 2017). The dopamine system, a major neuromodulatory input to the striatum has been consistently shown to be hyperactive in unmedicated TS patients (Wong et al., 2008; Palminteri et al., 2011). Functional neuroimaging investigations have been mostly conducted on the role of prefrontal and motor regions of the cortex, involved in cognitive control and movement generation, respectively. Studies provided evidence for the involvement of the motor, premotor and supplementer motor cortices in tic generation (Bohlhalter, 2006; Wang et al., 2011), along with increased activity in putative control regions of the cortex, such as dorsolateral prefrontal areas, in certain tasks (Baym et al., 2008; Ganos et al., 2014). This latter finding has been interpreted as being compensatory in nature and aiding the suppression of tics (Jackson et al., 2011). Indeed, evidence that cognitive control abilities have a causal role in tic reduction has been found (Yaniv et al., 2018). These lines of evidence altogether point to a disturbed structural and neurochemical profile of the basal ganglia, along with compensatory changes in the prefrontal cortex. Importantly, the involvement of these brain regions in procedural memory has been widely shown (Knowlton et al., 1996; Poldrack et al., 2001; Willingham et al., 2002; Fletcher et al., 2005; Ullman, 2015; Janacsek et al., 2020). Therefore, a better understanding of how procedural memory operates in TS could not only lead to a better characterization of its etiology but also to better behavioral and pharmacological treatment targets.

## Procedural Memory in Tourette’s Syndrome: Intact, Impaired or Enhanced?

We now review previous studies that investigated aspects of proceudral memory in TS (Table 1). An early study conducted by Kéri et al. (2002) used the weather prediction task, which requires probabilistic classification learning. The task involves learning to associate abstract cues in the shape of cards with weather outcomes. Importantly, the cues only have a probabilistic relationship with the outcomes, which is thought to disrupt declarative memorization strategies. This task has been prevously shown to activate the caudate nucleus (Poldrack et al., 2001; Foerde et al., 2006; Foerde and Shohamy, 2011). Kéri et al. (2002) found impaired classification performance in a sample of 20 children with TS and this impairment was more pronounced in children with more severe tics. Marsh et al. (2004) also employed the weather prediction task but with a more difficult probability structure, to study a large sample of 56 people with TS (32 children and 24 adults). They found similarly impaired classification accuracy in both adults and children with TS, and they also showed overall longer reaction times. Marsh et al. (2004) interpreted these signs of procedural impairment as a reflection of deficiencies in one of the proposed functions of the basal ganglia, namely the chunking of action sequences (Graybiel, 1998). According to this account, the striatal and dopaminergic abnormalities that occur in TS interfere with the typical firing patterns of neural assemblies in this region, which play a major role in habit learning and the chunking together of initially separate movements. This results in fragments of movements being executed independently and repetitively, manifesting at the behavioral level as tics.

**TABLE 1:** Summary of studies reviewed.

Study	Task	Sample	Medication^a^	Comorbidity^b^	Results
Kéri et al., 2002	WPT	20 TS; 20 controls	4 Antipsychotics	3 OCD; 4 ADHD	Weaker classification performance in TS
Channon et al., 2003	SRT	14 TS; 9 TS + ADHD; 6 TS + OCD; 21 controls	5 Antipsychotics; 2 antipsychotics + SSRI; 1 antipsychotics + stimulant; 2 SSRI; 2 antisymphatetic;1 tricyclic; 1 stimulant	See sample	Normal performance in all groups
Marsh et al., 2004	WPT	56 TS; 67 controls	3 Antipsychotics; 20 SSRI; 1 stimulant; 5 risperidone; 10 α-adrenergic agonist	13 OCD; 9 ADHD; 10 ADHD + OCD; 3 Depression; 8 ODD	Weaker classification performance in TS
Walenski et al., 2007	Past tense production; Picture naming	8 TS; 8 controls	1 Clonidine 9 risperidone; 1 clonidine + risperidone + clonazepam; 1 clonidine + dextroamphetamine; 1 clonidine + atomoxepine; 1 methylphenidate	1 ADHD; 1 ADHD + OCD	Faster past tense production of consistent regular verbs, regularized past tenses of novel verbs, over-regularization errors to irregular verbs; Faster naming pictures of manipulated objects; Lower accuracy on consistent regular past tense production in TS
Palminteri et al., 2011	Motor learning with reinforcement	20 Unmedicated TS; 15 medicated TS; 53 controls; 17 dystonia	In “unmedicated” group: 2 venlafaxine; 2 alprazolam; 1 clonazepam; 1 propanolol; 1 insulin In medicated group: 7 aripiprazole; 4 risperidone; 2 pimozide; 1 haloperidol; 1 cyamemazine; 1 tetrabenazine; 1 tiapride; 1 olanzapine; 2 venlafaxine; 1 valium; 1 lithium carbonate; 1 topiramate; 1 duloxetine	8 OCD	Decreased motor skill learning in all patient groups; Enhancement of reinforcement learning in unmedicated TS; Decreased reinforcement learning in medicated TS
Delorme et al., 2016	RL	17 Unmedicated TS; 17 medicated TS; 17 controls	In medicated group: 12 apiprazole; 2 pimozide; 1 risperidone; 2 mixed antipsychotics	4 OCD	Normal reinforcement learning in all patient groups; Increased levels of habitual responding in unmedicated TS
Dye et al., 2016	Non-word repetition	13 TS; 14 controls	1 Haloperidol + fluvoxamine; 1 aripiprazole; 1 lamotrigine + fluvoxamine; 1 dextroamphetamine + clonidine; 1 setraline hydrochloride; 1 risperidon; 1 pimozide + clonidine	3 OCD; 2 ADHD	Normal accuracy, but faster responses
Shephard et al., 2016	RL	18 TS; 13 ADHD; 17 TS + ADHD; 20 controls	In TS group: 2 clonidine; 1 fluoxetine + clonidine; 1 aripiprazole; 1 citalopram In TS + ADHD group: 1 clonidine + methylphenidate; 1 methylphenidate; 2 aripiprazole; 1 fluoxetine	See sample	Normal reinforcement learning in TS; Lower accuracy and impaired reversal learning in ADHD
Takács et al., 2017	ASRT	13 TS; 13 ADHD; 22 TS + ADHD; 21 controls	Unknown	See sample, additionally 1 eating disorder	Normal accuracy and reaction times in all groups
Takács et al., 2018	ASRT	21 TS; 21 controls	No medication	1 OCD; 5 ADHD	Normal reaction times, but increased accuracy in TS
Shephard et al., 2018	SRT	18 TS; 13 ADHD; 17 TS + ADHD; 20 controls	2 Clonidine; 1 clonidine + fluoxetine; 2 aripiprazole; 1 citalopram	See sample, additionally 3 OCD; 5 OCB; 3 Depression; 1 anorexia; 1 anxiety disorder	Normal sequence learning, but difficulty in transitioning in TS; Lower accuracy in ADHD
Tóth-Fáber et al., 2021	ASRT	21 TS; 21 controls	No medication	3 ADHD; 1 ADHD + OCD	Decreased sequence, but enhanced statistical learning in TS

*TS, Tourette’s syndrome; OCD, Obsessive-Compulsive Disorder; OCB, obsessive-compulsive behaviors; ADHD, Attention Deficit Hyperactivity Disorder; WPT, weather prediction task; SRT, serial reaction time task; ASRT, alternating serial reaction time task; RL, reinforcement learning.*

*^a^The medication status is given with the precision of the original article. However, it only includes the medication status of the TS group(s).*

*^b^Comorbidity is given with the precision of the original article. However, it only includes the comorbid disorders of the TS group(s).*

Further findings that challenged the impaired procedural functioning view were obtained by Channon et al. (2003), who studied 14 children with TS alone, 9 with comorbid TS and ADHD and 6 with comorbid TS and OCD, and controls. They administered the serial reaction time (SRT) task, which requires subjects to learn to respond to a visually presented sequence with the corresponding keys. After a number of such sequence blocks, random blocks without the previously learnt sequental structure are inserted. The performance decreases observed in this random blocks are thought to be indices of a primarily basal ganglia dependent form of learning. All patient groups performed all implicit and explicit memory measures at the same level as controls, including the SRT. Takács et al. (2017) used a modified SRT task to examine procedural learning in 13 children with TS only, 20 with comorbid TS and ADHD, 22 with ADHD only, as well as 21 controls. They found that children in all groups showed similar learning performance, both in terms of accuracy and reaction time learning measures.

Even more interestingly, more recent studies demonstrated not only intact but actually enhanced procedural learning. Takács et al. (2018) used the same sequence learning task as Takács et al. (2017) and found no differences between a sample of 21 TS patients and control participants on reaction time measures of learning. However, TS patients showed signs of markedly increased sequence knowledge on accuracy, especially at the end of the learning phase and during retest after a 16 h delay period. Shephard et al. (2018) also examined SRT performance in 18 children with TS, 13 with ADHD and 17 with comorbid TS and ADHD, along with controls. They found overall similar reaction times and accuracy in TS and control groups, with ADHD patients showing somewhat lower overall accuracy. There were differences, however, in how the different groups reacted to the disruption of learning when faced with stimuli without the previously learnt sequential structure. TS groups’ performance was considerably less disrupted by the inclusion of random blocks than the other groups’ which, according to Shephard et al. (2018), could indicate that the TS group overlearned the sequence.

Procedural enhancement is also obtained in experiments in multiple linguistic domains. Morphological processing enhancements were reported by Walenski et al. (2007), who examined past tense production and picture naming in 8 children with TS and 8 controls. They observed faster past tense production in TS of consistent regular verbs (e.g., slip–slipped), regularized past tenses of novel verbs (e.g., splim–splimmed) and over-regularization errors to irregular verbs (e.g., bring–bringed). TS children were also faster in the picture naming task in naming manipulated objects, but not in naming non-manipulated objects. In the case of past tense production, these reaction time increases were also accompanied by somewhat lower accuracy, suggesting a speed-accuracy tradeoff. These results are consistent with a generally faster processing of rule-based grammar in the procedural system. The findings were later extended to the phonological domain by the same group (Dye et al., 2016). They assessed the rule-based deconstruction of phonological strings using the non-word repetition task, which was administered to 13 children with TS and 14 control children. No difference in accuracy was found, however, TS children showed speeded production of non-words, suggesting enhanced procedural functions.

Evidence in support of increased habit learning comes not just from procedural memory, but also from reinforcement learning research. Reinforcement learning, the learning of correct actions based on environmental feedback in the form of rewards, also has been proposed to play a major role in the acquisition of habits. There is a high degree of similarity between the kinds of actions whose acquisition is examined in procedural learning studies and the habitual behaviors that are the focus of certain reinforcement learning studies (Graybiel, 2008). Research of reinforcement learning in TS has largely focused on the effects of dopaminergic medication and yielded results suggesting a dopaminergic hyperactivity and a corresponding deficit in learning from punishments in unmedicated TS patients (reviewed by Palminteri and Pessiglione, 2013). According to the hyperdopaminergia view, tics could be the result of an underactive negative reinforcement system, rather than an overactive positive one. Results pertaining to habit learning more strictly are those of Palminteri et al. (2011), who obtained evidence indicating increased effects of reinforcement in unmedicated TS patients on a sequence learning task, where stimuli were associated with different outcomes: high reward or minimal reward. While participants with TS showed enhanced learning in the high reward condition, the group difference was reversed when only minimal reward was present, suggesting distinct profiles of reinforcement and sequence learning in TS. This effect was abolished by dopamine agonist medication, indicative of an important role of dopamine signaling. A later study by Delorme et al. (2016) found increased levels of habitual responding in a slip-of-action test, after reinforcement learning. This increased engagement in habitual behavior in TS patients also correlated with greater structural connectivity within the right motor cortico-striatal network. Other work, however, did not find altered reinforcement learning. Shephard et al. (2016) used a simple reinforcement learning task and found normal performance and normal electrophysiological responses in TS, but impaired performance and altered ERPs in ADHD patients.

All in all, both procedural memory and reinforcement learning studies have provided mixed results regarding the establishment of procedures and habits, with a growing number of recent results suggesting increased learning/memory performance in TS. Intact procedural memory would simply imply that this system is largely unaffected in TS, but how might we account for such enhancements? The most likely explanation in the literature is that tics are the result of an overactive procedural system, that learns and produces even maladaptive movements automatically (Shephard et al., 2018). According to this view, the striatal and dopaminergic abnormalities in TS result in unnecessarily strong activity in the Go pathway of the basal ganglia, relative to the NoGo pathway (Maia and Frank, 2011). This might help procedural learning of new actions but also causes certain movement patterns to be executed even in environments that they are not suited to, becoming tics. The altered distribution and number of striatal interneurons also support this theory, as these neurons typically play a role in setting the excitation/inhibition balance to suitable levels in the striatum (Rapanelli et al., 2017).

## A Process-Based View of Procedural Memory

From this short review of the existing literature, it is clear that while a number of studies investigated procedural memory and reinforcement learning in TS, the findings have been rather mixed, with evidence for impaired (Kéri et al., 2002; Marsh et al., 2004), intact (Channon et al., 2003; Takács et al., 2017) and even enhanced functions (Walenski et al., 2007; Dye et al., 2016; Takács et al., 2018). Both a procedural hyperfunctioning and a procedural impairment account could be consistent with the behavioral symptoms and neurobiological underpinnings of the disorder. The diversity of the samples in both age and tic severity, the wide array of tasks used, the low sample sizes, the possible confounding effects of other cognitive functions, such as executive functions, working memory or attention all probably play some role in accounting for the heterogenous results. However, we propose that another, often overlooked factor, namely the multiprocess nature of the procedural system itself, could also significantly contribute to the varied pattern of empirical results.

It has been long recognized that a simple one-to-one correspondence between experimental measures and cognitive processes is highly unlikely; instead, tasks likely recruit multiple cognitive processes (Jacoby, 1991). Even the simplest cognitive operations require multiple, separate computational steps (Sigman and Dehaene, 2005). Moreover, in memory research specifically, it has also been suggested that, instead of distinguishing memory systems based on consciousness, there should be a greater emphasis on the processing modes during memory formation and retrieval (Henke, 2010). Process-oriented approaches of learning and memory (Foster and Jelicic, 1999) highlight that multiple processes underlie learning/memory tasks. Focusing on these processes and their interactions could help gain deeper insights into human memory and its neural underpinnings. Therefore, we propose that the study of procedural memory and its clinical relevance could benefit from a more process-oriented framework as well, rather than simply the use of singular performance metrics of complex tasks. This proposal is supported by two lines of argumentation below. Firstly, we show that there is a great degree of heterogeneity in the neural regions that are recruited during procedural learning, and that this heterogeneity reflects the involvement of multiple processes. Secondly, we highlight how a number of recent theoretical models on the functional level have also been built around the notion of multiple, distinct and interacting processes in various domains related to the detection of environmental regularities.

The involvement of the basal ganglia in procedural memory is well established (Ullman et al., 2020). Both the striatum and the putamen have been shown to be consistently activated by many different tasks that require learning predictive regularities, such as sequence learning (Janacsek et al., 2020), category learning (Foerde and Shohamy, 2011) or rhythm perception (Grahn and Rowe, 2009). Importantly however, even only considering the basal ganglia, variability arises due to the fact that the engagement of basal ganglia subregions seems to depend on the learning phase. Anterior and mid regions of the basal ganglia seem to be involved in the earlier phases of procedural learning and posterior regions appear to be more active as learning progresses (Jueptner et al., 1997a,b; Lehericy et al., 2005). Anterior basal ganglia is likely needed for the initial learning that is informed by more explicit, cognitive strategies, and as automaticity becomes greater, posterior basal ganglia takes the leading role in forming a more response based, motor sequence (Doyon et al., 2009). Furthermore, even though the ventral striatum is more robustly associated with reward based reinforcement learning (Graybiel, 2008), studies such as Palminteri et al. (2011) and Delorme et al. (2016), suggest that we would be wise to consider interactions between these processes and procedural ones. Moreover, while the basal ganglia are crucial, significant cortical engagement is also often found. In motor sequence learning, the primary motor cortex (M1) also seems to have an important role (Steele and Penhune, 2010, but see Berlot et al. (2020) for a reevalutation of these findings). This region is directly responsible for voluntary movement generation and is a good candidate for the storage of motor representations. Its involvement in procedural memory then would be the storage of the motor components of tasks, whose execution would be informed by the regularities learnt by the basal ganglia. Influential models of basal ganglia function have proposed that these regions actually teach cortico-cortical connections (e.g., in the motor domain, those of M1), and after sufficient time and training, task execution will become reliant on these connections instead, and be relatively independent of basal ganglia (Ashby et al., 2010; Hélie et al., 2015). The cerebellum is another area that is commonly found to be engaged in procedural learning (Steele and Penhune, 2010; Janacsek et al., 2020). Penhune and Steele (2012) proposed that the functional role of this region in motor sequence learning is the coding of internal forward models that predict the sensory consequences of actions, and help error correction. The role of this structure might also extend to non-motor domains. For example Janacsek et al. (2020) highlight that the cerebellar region they found to be systematically engaged by sequence learning could serve explicit knowledge and spatial working memory. Explicit knowledge, the intentional search for patterns and the use of working memory (Janacsek and Nemeth, 2013) could also explain the recruitment of prefrontal regions, that has sometimes been observed in some procedural memory studies as well (Willingham et al., 2002; Fletcher et al., 2005). If we focus our attention now to perception, we find further regions. In statistical learning, which can be considered as a form of purely perceptual procedural learning, in addition to the regions already examined, we find evidence for the involvement of more sensory modality specific brain regions (Frost et al., 2015). These include the cuneus and the fusiform gyrus for visual and the superior temporal gyrus for auditory statistical learning. Finally, the exact function of the medial temporal lobe (MTL) and the hippocampus in regularity detection is heavily debated. Earlier studies provided evidence for its necessity for statistical learning (Schapiro et al., 2014), however, recent results have challenged this view (Rungratsameetaweemana et al., 2019). The involvement of the MTL does not seem to be related to the explicitness of the representation (Schendan et al., 2003). One proposal has been that while the basal ganglia are involved in the learning of representations in egocentric space, the role of the MTL is to learn allocentric representations in parallel (Albouy et al., 2015). In summary, we can observe a significant amount of variability in the neural regions that underlie procedural memory, implicating a distributed brain network, with each region contributing different processes to the overall function of using environmental regularities for optimizing actions.

If we now turn to the functional level, we again find that several theoretical frameworks have already been proposed to describe the multiple, distinct, interacting processes underlying various non-declarative learning tasks. For example, in statistical learning, one distinction has been introduced between mechanisms that extract perceptual units from the environment and mechanisms that integrate over extracted units (Thiessen et al., 2013). In this distinction, the former process acquires transitional statistical information, while the latter acquires distributional statistical information. Various studies in both the auditory and visual modalities support this two process view of learning (De Diego Balaguer et al., 2007; Endress and Bonatti, 2007; Mirman et al., 2010; Zhao et al., 2011; Wang et al., 2019). A more recent model by Conway (2020) also distinguishes two, interacting mechanisms that underlie statistical learning. A more implicit, modality specific, automatic mechanism that is sensitive to simple, surface level structures and a more explicit, domain general, attention dependent mechanism, that can also acquire more complex structural relationships. Additionally, in deterministic sequence learning, Beukema and Verstynen (2018) proposed a model with a fast associative learning of transitional structure that supports predictions, along with a slower binding of these individual transitions into sets. The former has been proposed to rely on the MTL and the latter on the basal ganglia. Finally, Maheu et al. (2020) recently made the case that the brain considers deterministic, rule-like predictions and statistical biases as two distinct hypothesis spaces, that are linked by a common probabilistic currency. A pattern seems to emerge from all of these models, according to which the brain employs at least two distinct sets of processes during the learning of tasks that require the extraction of environmental regularities: a set of learning processes sensitive to statistical biases and a set of learning processes sensitive to deterministic sequences. However, this hypothesis is greatly speculative as the described theoretical models have been proposed to account for different kinds of tasks. What seems clear, however, is that many of these proposed learning mechanisms in various domains likely operate dynamically, both in a parallel and hierarchical manner, and make distinct contributions to performance in procedural memory tasks.

Overall, based on the arguments discussed above, we believe that taking a process-based approach could greatly contribute to a deeper understanding of procedural memory both in basic and clinical research. A recent review by Bogaerts et al. (2020) made similar points with respect to the study of statistical learning deficits in language disorders. They noted that there is a multiplicity of both theoretical constructs and experimental measures, and this makes the results of individual studies difficult to reconcile. We share these concerns and believe that the use of process-level theoretical constructs could help mitigate them, both in TS research and procedural memory research more broadly. If procedural memory is considered as a bundle of interacting processes instead of a single system, it seems likely that different tasks will involve these processes to different degrees. Then it is possible that, instead of enhanced or impaired performance, a more complex pattern will emerge, with decreased effectiveness in some processes and increased in others.

## Methodological Considerations and Examples of Process-Based Approaches in Procedural Memory

In order to truly employ the framework we propose, we need to have adequate methods that make it possible to investigate multiple learning mechanisms at once. Considering the online or offline nature of the measure is of great importance in this regard (Siegelman et al., 2017). Online, processing based measures assess learning performance during task execution and are sensitive to the temporal dynamics of learning. Some versions of the SRT or the visuomotor adaptation task are online measures. Offline, reflection based measures assess learning and memory performance after learning has occurred. Temporal dynamics might be particularly useful for understanding the dissociable, parallel mechanisms that might operate during procedural memory formation. Results from an offline procedure would likely reflect the contribution of all processes. Furthermore, online measures have been proposed to be more reliable, less confounded by interference effects and to better reflect individual differences (Siegelman et al., 2017).

The use of probabilistic, online sequence learning tasks could alleviate the reliability concern and lead to a clearer picture. The alternating serial reaction time (ASRT) task is a variant of the SRT paradigm, which intersperses pattern stimuli with random ones, resulting in a complex, probabilistic structure with second-order non-adjacent dependencies (Howard and Howard, 1997; Nemeth et al., 2010). The task has been shown to be more reliable and sensitive to individual differences (Stark-Inbar et al., 2017). Furthermore, ASRT allows the study of the temporal trajectory of learning. The task also seems to be capable of disentangling the role of multiple processes that operate during procedural learning. For example, Nemeth et al. (2013) found that a process termed *statistical learning* was evident in learners’ better performance for stimuli that were more predictable than for stimuli that were less predictable based on the *n*-2 stimulus, even though both the more and less predictable stimuli appeared in random positions. A complementary process called *sequence learning* was evident in learners’ better performance for stimuli that followed a deterministic sequence pattern than for stimuli that appeared in the random positions, even though both were equally predictable based on the *n*-2 stimulus. Thus, in this task, statistical learning refers to the acquisition of shorter-range relations between stimuli based primarily on probabilistic (predictability-based) information. This information seems to be learned relatively rapidly and incidentally (i.e., without the intention to learn and awareness that learning occurs) (Kóbor et al., 2018; Simor et al., 2019). Sequence learning refers to the acquisition of order-based information while predictability-based characteristics are equal between the compared elements; in other words, participants learn a series of repeating elements occurring in the same order with embedded noise between them. Sequence learning can occur both in incidental and intentional learning situations, with typically faster learning in the intentional learning condition (Howard and Howard, 1997; Howard et al., 2004; Nemeth et al., 2013; Simor et al., 2019). It is interesting to note the similarity between the two processes uncovered in this task and the two sets of processes that seem to fall out of the theoretical frameworks discussed above. Sequence learning resembles closely the rule-based processes, whereas statistical learning resembles the probabilistic processes of Maheu et al. (2020). Importantly, the contribution of these two, seemingly distinct processes is mixed together if the researchers only use the standard metric of the ASRT task, the performance difference between high-probability and low-probability stimulus triplets.

In subsequent studies, statistical and sequence learning were found to be dissociable based on the learning trajectories they show during task execution (Simor et al., 2019; Quentin et al., 2021) and electrophysiological correlates (Kóbor et al., 2018; Zavecz et al., 2020; Takács et al., 2021) as well. On the behavioral level, sequence learning seems to increase gradually during training, while statistical learning seems to plateau early (Kóbor et al., 2018; Simor et al., 2019). Moreover, it has been suggested that sequence learning is acquired offline during rest periods, while statistical learning occurs online during the task execution (Quentin et al., 2021). ERP components also reflected a distinct trajectory of sequence and statistical learning (Kóbor et al., 2018). Moreover, Simor et al. (2019) have found differences in neural oscillations during consolidation between statistical and sequence learning. Slow frequency oscillations (high delta and theta power) during sleep predicted further improvements in sequence learning, while changes in statistical learning were not associated with spectral EEG power measures. The two mechanisms also have distinct functional connectivity patterns during consolidation (Zavecz et al., 2020) and seem to be associated with different aspects of information coded in the N2 time window (Takács et al., 2021).

A study using this paradigm has previously provided support for a procedural hyperfunctioning in TS (Takács et al., 2018), however, the task has not been widely used to study multiple learning processes at once in the disorder. Recently, a study by Tóth-Fáber et al. (2021) has attempted to fill in this gap and used the ASRT task to separate statistical and sequence learning processes in TS. They administered the task to 21 TS patients and 21 age, gender and education matched controls. The results suggested an impairment in sequence learning, as patients showed evidence of this process only in the very beginning of the task. Statistical learning, on the other hand, seemed enhanced compared to controls, especially during the early period of learning. Thus, it seems that TS patients could have impairments in the acquisition of order-based, transitional regularities, while they could be more sensitive to probabilistic information. This is consistent with the nature of tics, which look like the result of a mechanism that produces certain movements more frequently than needed, with the movements being fragmented and not conforming to the typical transitional structure of actions. The fact that these two processes were differently and independently affected in the disorder suggests that these processes are supported by separate computations in the brain. Moreover, it is also suggestive that considering the multiprocess nature of procedural memory could be an important step toward the understanding of the neurocognitive profile of TS.

Neuroimaging methods can also be extremely useful in disentangling different processes that might underlie the same behavior. A recent study by Beste et al. (2021) illustrates this. Working in the theory of event coding framework, they administered a perception-action integration task to 32 TS patients and 27 typically developing controls. While behaviorally, the groups did not differ as both showed robust binding effects, there were pronounced differences in the underlying patterns of neural oscillations. Specifically, during perception-action integration, theta band activity was localized to superior frontal regions, including the supplementary motor area (SMA), in typically developing controls, whereas in TS patients, the distribution of theta activity was more pronounced in parietal and inferior frontal regions. This led the authors to hypothesize that in TS, the processing resources of the SMA are limited by its tic-related activity, leading to the recruitment of different neurocognitive mechanisms during perception-action integration. Such results were only possible to obtain by looking beyond standard behavioral indices.

It is also possible to find examples where a process-level analysis has led to important insights in the study of attentional and executive systems. While not focusing on procedural memory, a recent study by Veríssimo et al. (2021) has highlighted the value that a process-level decomposition can bring to the study of neurocognition and partially reconcile contradictory findings. They administered the ANT task, a task with high levels of reliability and validity to a large sample of participants aged between 58 and 98 to shed light on the trajectory of attentional network efficiency with aging. Similarly to our case of procedural memory in TS, previous reports of attentional changes in aging have been mixed, with studies showing declines, stagnation and even improvements. Importantly, the use of the ANT enabled the decomposition of the broad construct of attention to three well established components, alerting, orienting and executive inhibition. The results have shown dissociable effects in the three networks, with alerting declining, the other two networks improving with age. These results, which partially account for the earlier mixed findings, would not have been possible to obtain without a process-level decomposition.

In the domain of executive functions, a study by Yaniv et al. (2017) similarly highlights the importance of process-level analysis, this time, specifically in TS. They too aimed to reconcile conflicting earlier reports of executive functioning in TS, by using a more precise approach and decomposing the executive system to inhibition, set shifting and updating (Miyake and Friedman, 2012). They administered a neuropsychological battery, consisting of the Wisconsin Card Sorting Task, the Stop Signal Task and a previously validated Task switching task (Tayeb and Lavidor, 2016). They analyzed both individual performance measures and compositions of these from a factor analysis. The results suggested that the primary and specific impairment in TS is in the inhibitory component.

Overall, based on existing evidence thus far, it seems likely at least some of the variability in procedural memory performance reported in the literature could be due to tasks engaging different sets of processes, including different learning processes. While we limit our review to procedural memory specifically, we believe that our framework generalizes to other cognitive domains, such as executive functions or attention. Research in these fields could also benefit from process-level analysis, as numerous recent studies have demonstrated, both in TS (Yaniv et al., 2017) and in other populations of interest (Veríssimo et al., 2021). The separation of distinct computations engaged during learning through computational modeling and the use of online measures will likely be crucial to understand how procedural memory operates in health and disease.

## Conclusion and Future Directions

In this brief review, we presented the current state of the evidence for altered procedural memory and habit learning in Tourette’s syndrome. Impaired (Kéri et al., 2002; Marsh et al., 2004), intact (Channon et al., 2003; Takács et al., 2017) and enhanced (Dye et al., 2016; Takács et al., 2018) learning and memory performance were reported by previous studies. Both hypoactive and hyperactive procedural learning oriented theories could be consistent with the underlying neurobiology and behavioral symptoms of the disorder. We argued that adopting a process-based view of memory, consistent with recent proposals in the literature, could lead to more consistent results and a deeper understanding of neurocognitive underpinnings of procedural memory in health and disease. Shifting the focus of research from memory systems to processes might reveal the complex pattern of impairments and enhancements that presents itself in Tourette’s syndrome and neurodevelopmental disorders more generally. These would otherwise be hidden behind performance measures that average together the unique contributions of a range of processes.

We emphasized that the decomposition of tasks into distinct computational steps and strategies the performers might engage in is crucial for a thorough understanding (Hunt and Aslin, 2001). Different learning processes that extract environmental statistics and action patterns should be distinguished within procedural memory tasks. Beyond the type of regularity being encoded (Thiessen et al., 2013), the timescale of the integration needed to learn the regularity (Maheu et al., 2019), whether the regularity consists of adjacent or non-adjacent dependencies (Wang et al., 2019), the modality the stimuli are presented in Conway and Christiansen (2006) and whether linguistic or non-linguistic stimuli are used (West et al., 2018) could all influence the processes that are engaged in procedural memory. These factors might also differentially affect the consolidation of the acquired knowledge of the different regularities (Cohen et al., 2005). Therefore, it is unlikely that impaired or enhanced functioning of procedural memory as a whole could underlie any disorder. As discussed above, some recent studies that separated multiple learning processes in a single domain have already provided some interesting results in Tourette’s syndrome (Yaniv et al., 2017; Beste et al., 2021; Tóth-Fáber et al., 2021). Furthermore, the components could also have distinct relationships with other cognitive functions, for example they might depend more or less on working memory capacity, executive functions or attentional resources (Janacsek and Nemeth, 2013, 2015; Otto et al., 2013a,b). Finally, we argued the use of online measures could be especially important in the effective application of the process-based view.

While we restricted our discussion to Tourette’s syndrome, it is also tempting to extend our suggestions to other disorders and even typical development. A dysfunctional procedural memory system has also been proposed to account for neurodevelopmental disorders affecting language, such as Dyslexia or Developmental Language Disorder (Ullman and Pierpont, 2005; Ullman et al., 2020). If procedural memory is indeed supported by a multitude of processes, then further investigation should focus on the exact computations and environments in which impairments or enhancements can be found. The same holds for typical development. For example, it has been suggested that at least some aspects of procedural learning ability peaks during adolescence and declines through adulthood (Nemeth et al., 2013; Juhasz et al., 2019; Zwart et al., 2019, but see Lukács and Kemény, 2015). If multiple, separate cognitive processes contribute to procedural learning in the tasks that are typically used to assessed it, then those components may be differently affected by age (Nemeth et al., 2013). It could thus be extremely worthwhile to examine the maturation of putative processes involved in procedural memory and to understand their role in individual differences of procedural memory (Kaufman et al., 2010; Frost et al., 2015; Siegelman et al., 2017).

A major gap in this line of inquiry is the lack of longitudinal studies that could shed light on the causal relationships between procedural learning mechanisms and clinical symptoms. Currently very little data is available on how the neuropsychological and neurobiological profile of Tourette’s syndrome changes across the lifespan and how these factors interact with tic severity, premonitory urges or other clinical constructs of interest. This is even more pertinent given results described above that indicate differential developmental trajectories for different aspects of procedural memory (Lukács and Kemény, 2015; Juhasz et al., 2019; Zwart et al., 2019). In conclusion, we think that while the use of higher-order constructs like memory systems is still appropriate, it is time to complement this by considering the role that lower level computations and processes play both in Tourette’s syndrome and neurodevelopment in general.

## Author Contributions

BF: conceptualization, writing – original draft, review, and editing. ET-F: conceptualization, writing – review and editing. KJ: writing – review and editing. DN: conceptualization, writing – review and editing, supervision, and funding acquisition. All authors contributed to the article and approved the submitted version.

## Conflict of Interest

The authors declare that the research was conducted in the absence of any commercial or financial relationships that could be construed as a potential conflict of interest.

## Publisher’s Note

All claims expressed in this article are solely those of the authors and do not necessarily represent those of their affiliated organizations, or those of the publisher, the editors and the reviewers. Any product that may be evaluated in this article, or claim that may be made by its manufacturer, is not guaranteed or endorsed by the publisher.
